# Effects of dietary energy density and feeding strategy during the dry period on feed intake, energy balance, milk production, and blood metabolites of Holstein cows

**DOI:** 10.3168/jdsc.2022-0233

**Published:** 2022-08-06

**Authors:** A. Pineda, F.C. Cardoso, M.R. Murphy, J.K. Drackley

**Affiliations:** 1 Department of Animal Sciences, University of Illinois, 1207 West Gregory Drive, Urbana 61801

## Abstract

Our study aimed to assess the effects of dietary energy density and strategy of delivery during the dry period on production and metabolic responses of Holstein cows free of displacement of abomasum, retained placenta, metritis, or hypocalcemia. Twenty-seven multiparous cows dried-off 50 d before calving were assigned randomly to 1 of 3 dietary treatments: a controlled energy, high-fiber diet [CE; 1.39 Mcal net energy for lactation (NE_L_)/kg of dry matter (DM)] formulated to supply 100% of the NE_L_ requirement at ad libitum intake; or a higher energy diet (1.58 Mcal/kg) fed either at ad libitum (HE) or restricted (RE) intake to supply ∼150 or 80% of the NE_L_ requirements, respectively. After calving, all cows were fed the same lactation diet. Cows were individually fed and remained in the study until 28 d postpartum. Data were analyzed using 2 contrasts: CE versus HE (effect of diet composition in cows fed for ad libitum DM intake) and HE versus RE (effect of ad libitum or restricted intake of the same diet). Prepartum intakes of DM and NE_L_ as well as energy balance (EB), were greater for HE than CE and greater for HE than RE. Body weight (BW) gain was higher in HE than in RE, but CE and HE did not differ. Change in body condition score did not differ between CE and HE or HE and RE. Postpartum intakes of DM and NE_L_, EB, BW, body condition score, calf birth BW, milk yield, and milk components did not differ between CE and HE or HE and RE. Concentrations of glucose, insulin, nonesterified fatty acids, β-hydroxybutyrate, Ca, and Mg pre- and postpartum did not differ among diets. Although sample size was low, dry period plane of energy intake affected prepartum DM intake, EB, and BW gain but did not affect postpartum intakes of DM and NE_L_, yields of milk and milk components, or blood metabolites in healthy cows.

## Highlights

• High energy diets fed ad libitum before calving increased intakes and energy balance compared with cows fed a lower energy diet or restricted amounts of a high energy diet.
• Prepartum diets did not affect postpartum intakes or production in healthy cows.
• Prepartum diets did not affect postpartum blood metabolites.

## Graphical Abstract

**Figure:**
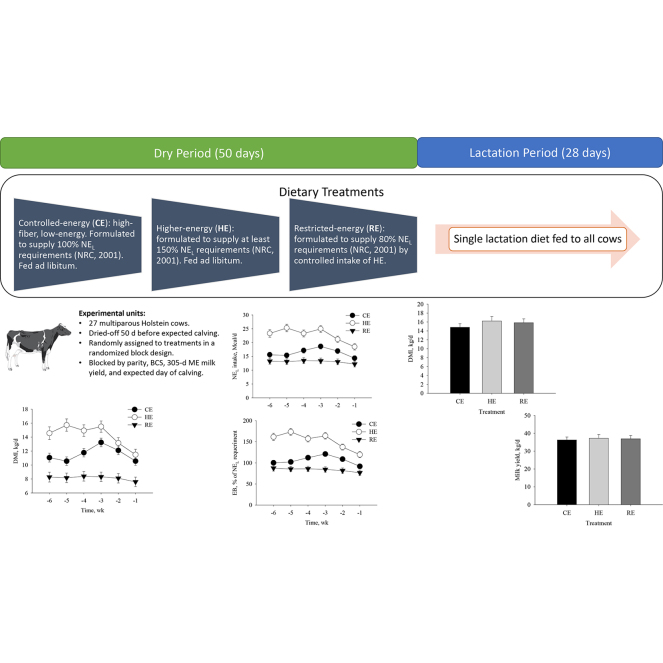
**Summary:** Dietary plane of energy intake during the dry period significantly affected dry matter intake, energy balance, and body weight gain, but did not affect body condition score or its change in cows that were free of displacement of abomasum, retained placenta, metritis, and hypocalcemia. Prepartum energy intake did not affect postpartum intakes of dry matter and energy, milk yield, milk composition, or blood metabolites measured 5 d before through 5 d after calving.

Nutritional management strategies during the transition period aim to promote greater postpartum DMI (and energy intake) and modulate fat mobilization. Strategies include moderate or high energy density diets fed for ad libitum intake (VandeHaar et al., 1999; Rabelo et al., 2003) and controlled energy (Cardoso et al., 2013) either by high-fiber, low-energy diets fed at ad libitum intake (Janovick and Drackley, 2010; Mann et al., 2015; Richards et al., 2020) or by restricted feeding (Dann et al., 2006; Douglas et al., 2006; Winkelman et al., 2008). Although controlled studies recorded disease incidents, most were underpowered to assess accurately the effects of dry period treatments on true disease incidence. Instead, effects were inferred from changes in blood nonesterified fatty acids (**NEFA**), BHB, and Ca. Controlled energy programs that maintained energy intake near requirements, either by feeding bulky low-energy forage (Dann et al., 2006; Janovick et al., 2011; Richards et al., 2020) or by restricting feed intake (Dann et al., 2006; Douglas et al., 2006; Janovick et al., 2011), decreased NEFA and BHB compared with high energy diets. In a pooled statistical analysis, Cardoso et al. (2013) found that controlled energy programs decreased the risk of displaced abomasum and clinical ketosis. A significant question remaining unanswered is whether controlled energy programs alter metabolism in all cows in a way that results in greater productivity or whether they somehow decrease the incidence of peripartal health problems, which leads to greater productivity.

Our study aimed to assess the effects of a controlled energy diet fed for ad libitum intake or a higher energy diet fed for ad libitum or restricted intakes on blood metabolites before and after parturition, as well as DMI, energy balance, and milk production. Data from Holstein cows free of displacement of abomasum, retained placenta, metritis, and hypocalcemia were used to avoid the confounding effects of these disorders on production and metabolic responses. Our hypothesis was that cows fed the ad libitum controlled energy regimen would have improved peripartal outcomes (i.e., decreased NEFA and BHB, increased postpartum DMI and milk production) compared with cows fed high energy or restricted intake regimens, and that this would be independent of differences in disease outcomes.

The University of Illinois Institutional Animal Care and Use Committee approved all procedures (protocol 11193). Multiparous pregnant Holstein cows (n = 38) were blocked regarding parity (3.1 ± 1.1, mean ± SD), previous lactation 305-d mature-equivalent milk yield (12,015 ± 1,509 kg), BCS (3.5 ± 0.4), and expected day of calving. Within blocks, cows were randomly assigned to 1 of 3 dry period diets in a randomized incomplete block design. Dietary treatments were (1) controlled energy, high-fiber diet (**CE**, n = 11) to supply 100% of NRC (2001) requirements for NE_L_ and all nutrients when fed for ad libitum intake; (2) high energy diet (**HE**, n = 7), fed for ad libitum intake to supply at least 150% of NE_L_ requirements (NRC, 2001); and (3) restricted high energy diet (**RE**, n = 9), fed to supply 80% of the calculated NE_L_ requirement (NRC, 2001) by limited intake of the HE ration (Table 1). The amount of feed offered daily to RE cows was adjusted weekly according to the NRC (2001) equations, accounting for dietary DM and energy contents. Diets included MgCl_2_ and MgSO_4_ as bioavailable Mg sources that brought the DCAD to near zero. Cows remained on treatments from dry-off through parturition (CE = 48 ± 4 d; HE = 47 ± 5 d; and RE = 48 ± 4 d). A common lactation diet was fed to all cows after parturition until 28 DIM.

**Table 1:** Ingredient and nutrient composition of the diets fed to multiparous Holstein cows during the dry and early lactation periods

Item	Dry period diet^1^		Lactation diet
	CE	HE and RE	
Ingredient, % of DM			
Alfalfa hay	1.99	5.97	3.36
Alfalfa silage	8.88	13.6	9.32
Corn silage	33.2	54.1	33.6
Wheat straw	36.0	—	2.80
Cottonseed	—	4.98	8.02
Ground shelled corn	4.04	12.6	20.9
Soy hulls	—	—	4.66
Soybean meal, 48% CP	11.6	4.35	4.29
Expeller soybean meal^2^	—	—	8.39
Blood meal, 85% CP	—	—	1.30
Urea	0.20	0.19	—
Rumen-inert fat^3^	—	—	0.34
Limestone	0.82	0.84	1.07
Salt (plain)	0.20	0.14	0.21
Dicalcium phosphate	0.79	0.70	0.40
Magnesium chloride	0.46	0.70	—
Magnesium oxide	0.40	0.38	0.07
Magnesium sulfate	0.99	1.05	—
Sodium bicarbonate	—	—	0.74
Calcium chloride	—	—	0.12
Mineral-vitamin mix^4^	0.20	0.21	0.37
Vitamin A^5^	0.01	0.01	—
Vitamin D^6^	0.01	0.01	—
Vitamin E^7^	0.26	0.24	0.04
Nutrient content^8^			
DM, %	47.3 ± 3.13	43.4 ± 2.51	50.5 ± 3.72
CP, % of DM	14.3 ± 0.63	15.0 ± 0.46	18.1 ± 0.78
ADF, % of DM	31.1 ± 2.61	23.1 ± 2.62	21.6 ± 2.14
NDF, % of DM	45.7 ± 3.29	36.8 ± 5.91	32.4 ± 1.71
Lignin, % of DM	5.12 ± 0.79	4.32 ± 0.73	5.30 ± 2.18
Starch, % of DM	14.6 ± 3.68	23.3 ± 6.44	25.9 ± 2.52
Crude fat, % of DM	2.52 ± 0.12	4.00 ± 0.30	4.85 ± 0.41
NE_L_, Mcal/kg of DM	1.39 ± 0.06	1.58 ± 0.06	1.64 ± 0.06
Ca, % of DM	1.09 ± 0.28	0.96 ± 0.14	1.00 ± 0.06
P, % of DM	0.35 ± 0.04	0.42 ± 0.03	0.43 ± 0.01
Mg, % of DM	0.51 ± 0.03	0.59 ± 0.06	0.28 ± 0.03
K, % of DM	1.26 ± 0.11	1.27 ± 0.14	1.19 ± 0.11
Na, % of DM	0.26 ± 0.35	0.10 ± 0.01	0.27 ± 0.02
S, % of DM	0.28 ± 0.02	0.33 ± 0.02	0.24 ± 0.01
IVTD 30 h,^9^ % of DM	75.7 ± 1.63	83.7 ± 2.07	86.2 ± 0.98
NDFD 30 h,^10^ % of NDF	47.0 ± 1.79	55.2 ± 3.06	57.2 ± 3.06
DCAD, mEq/kg DM	88	63	252

1 CE = controlled energy to supply 100% of the NRC (2001) requirements for NE_L_ and all nutrients, fed ad libitum intake. HE = high energy to supply at least 150% of the NRC (2001) requirements for NE_L_, fed ad libitum intake. RE = restricted energy to supply 80% of the NRC (2001) requirements for NE_L_ by restricted intake of the HE diet.

2 SoyPLUS (West Central Cooperative).

3 Energy Booster 100 (Milk Specialties Co.).

4 Contained a minimum of 5% Mg, 10% S, 7.5% K, 2.0% Fe, 3.0% Zn, 3.0% Mn, 5,000 mg/kg of Cu, 250 mg/kg I, 40 mg/kg Co, 150 mg/kg of Se, 2,200 kIU/kg of vitamin A, 660 kIU/kg of vitamin D_3_, and 7,700 IU/kg of vitamin E.

5 Contained 30,000 kIU/kg.

6 Contained 5,009 kIU/kg.

7 Contained 44,000 IU/kg.

8 Nutrient composition (mean ± SD) based on 4-wk feed ingredient composites.

9 In vitro true digestibility at 30 h.

10 NDF digestibility at 30 h.

Thirty-eight cows completed the study. This study was part of a larger study that determined responses to glucose and insulin challenges (data reported elsewhere). For our purpose, we selected the 27 cows free of displacement of abomasum, retained placenta, metritis, and hypocalcemia. Displaced abomasum was detected by veterinary staff. Retained placenta was defined as placenta not released at 24 h after parturition. Metritis was defined as abnormal vaginal discharge. Hypocalcemia was defined as a cow in recumbency and responsive to i.v. Ca therapy. There were 6, 5, and 0 cows removed as nonhealthy from HE, CE, and RE groups, respectively. Power analysis indicated that 6 cows per treatment could detect a difference of 2.0 mg/dL in postpartum BHB concentration with 80% power; the power to detect differences in milk production was lower.

The experiment was conducted mostly during summer (May to October). The temperature-humidity index (**THI**) was calculated using ambient temperature and relative humidity from Illinois State Water Survey (http://www.isws.illinois.edu) data and the equation of Dikmen and Hansen (2009). Cows were housed in a freestall barn during the dry period and in an enclosed ventilated tiestall barn after parturition. Cows fed CE, HE, and lactation diets were individually fed 115% of expected intake at 0600 h daily. Water was available at all times. Body weight and BCS were measured once weekly. The BCS was assessed by 2 trained individuals using a 5-point scale in 0.25-point increments.

Dry matter intake was measured daily. The DM concentration of TMR and its components were determined weekly, so that TMR composition and amounts of dietary DM offered were adjusted weekly. The TMR and its components were sampled weekly, stored at −20°C, composited by month, and then analyzed by wet chemistry techniques for nutrient concentrations (Table 1) at a commercial laboratory (Dairy One, Ithaca, NY). Particle size distribution of the TMR offered and TMR refused was measured once weekly using the Penn State Particle Separator (Kononoff et al., 2003).

Cows were milked 3 times daily (0415, 1215, and 2015 h), and milk yields were electronically recorded. Milk samples were collected twice weekly and analyzed for fat and protein by mid-infrared spectroscopy at a commercial laboratory (Dairy Lab Services). Yields of 3.5% FCM, ECM, and milk components were calculated using daily milk yield and milk component concentrations. Daily samples of blood were obtained by puncture of a coccygeal vein or artery with 20-gauge × 2.5 cm needles (Becton Dickinson and Company) from d 5 before through 5 d after calving to detect differences in the highly variable changes around parturition. Samples were collected <1 h after feeding into evacuated tubes containing clot activator and K_2_EDTA (Becton Dickinson and Company) for serum and plasma, respectively. Samples for plasma were placed on ice immediately after collection. Samples for serum were allowed to clot at room temperature for at least 30 min and then placed on ice. All tubes were centrifuged, within 2 h of collection, at 4°C for 15 min at 959 × *g*. After centrifugation, aliquots of serum and plasma were kept at −20°C until analysis of glucose, insulin, and NEFA (Osorio et al., 2013). Concentrations of BHB, Ca, and Mg were determined at the University of Illinois College of Veterinary Medicine Clinical Pathology Laboratory using an AU680 Beckman Coulter Chemistry Analyzer (Beckman Coulter Inc.; https://vdl.vetmed.illinois.edu/clinical-pathology).

Statistical analyses was conducted using the MIXED procedure of SAS (v9.4, SAS Institute Inc.). The model contained the fixed effects of treatment, time, and their interaction, and the random effects of block and cow within block (experimental unit). Two predetermined contrasts were specified: CE versus HE (i.e., the effect of diet composition in cows fed for ad libitum DMI) and HE versus RE (i.e., the effect of ad libitum or restricted intake of the same diet). Residuals were assessed for normality and homoscedasticity. Statistical significance was declared at *P* ≤ 0.05, and trends toward significance when 0.05 < *P* ≤ 0.10.

The ingredient and nutrient composition of diets fed during the study is in Table 1. The DMI during the dry period differed (*P* < 0.01) among treatments (Table 2). Cows fed HE consumed 2.7 and 6.1 kg/d more DM than CE and RE, respectively. The HE cows had 21% greater DMI than CE because of the greater forage NDF content for CE (45.7% vs. 36.8% for CE and HE). We observed an interaction of treatment and time (*P* = 0.02) for DMI prepartum due to HE and CE having increased DMI during the first 4 wk following dry-off and a decrease in DMI 2 wk before parturition. For cows in RE, DMI remained constant throughout the dry period (8.1 ± 0.68 kg/d), consistent with previous studies (Winkelman et al., 2008; Janovick and Drackley, 2010). Decreases in DMI 2 wk before partition were more pronounced in HE and CE than in RE-fed cows (32, 20, and 8%, respectively).

**Table 2:** Least squares means for BW, BCS, DMI, and energy balance and milk components of cows fed controlled energy (CE), high energy (HE), or restricted energy (RE) diets during the dry period

Item	Treatment			SEM^1^	*P*-value			
	CE	HE	RE		Time	Treatment × time	CE vs. HE	HE vs. RE
Prepartum								
BW, kg	786	758	818	32.7	<0.01	0.19	0.51	0.18
BW change,^2^ kg	40.7	54.9	11.7	8.95	—	—	0.22	<0.01
BCS	3.57	3.49	3.59	0.12	0.03	0.88	0.65	0.56
BCS change^2^	−0.16	0.07	−0.08	0.12	—	—	0.15	0.35
DMI, kg/d	11.6	14.1	8.13	0.52	<0.01	0.02	<0.01	<0.01
NE_L_ intake, Mcal/d	16.3	22.7	13.1	0.86	<0.01	0.05	<0.01	<0.01
Energy balance, Mcal/d	0.90^b^	7.77^a^	−2.59^c^	0.84	<0.01	0.02	<0.01	<0.01
Energy balance,^3^ %	106	152	84	5.33	<0.01	0.01	<0.01	<0.01
Calf birth BW, kg	43.8	44.5	43.1	1.36	—	—	0.71	0.52
Postpartum								
BW, kg	664	650	706	28.5	<0.01	0.96	0.71	0.15
BCS	2.97	3.00	2.99	0.12	<0.01	0.55	0.87	0.93
BCS change^2^	−0.13	−0.39	−0.25	0.11	—	—	0.07	0.35
DMI, kg/d	14.8	16.2	15.8	1.00	<0.01	0.47	0.29	0.77
NE_L_ intake, Mcal/d	23.6	26.3	25.0	1.78	<0.01	0.24	0.27	0.59
Energy balance, Mcal/d	−16.7	−14.9	−14.6	1.53	0.01	0.38	0.38	0.91
Energy balance,^3^ %	58.3	64.4	61.6	3.06	<0.01	0.22	0.14	0.49

1 Greatest SEM.

2 For BW and BCS, prepartum change was calculated subtracting the last value before parturition minus the value at dry-off. Postpartum change was calculated subtracting the value at wk 4 minus the value at wk 1.

3 Expressed as a percentage of NE_L_ requirements.

Cows fed HE consumed 6.4 and 9.6 Mcal/d more NE_L_ than CE and RE (Table 2). Groups HE and CE consumed 7.8 and 0.9 Mcal/d above their NE_L_ requirements, whereas RE consumed 2.6 Mcal/d less than requirements, in agreement with previous results (Dann et al., 2006; Douglas et al., 2006; Janovick and Drackley, 2010). According to NRC (2001), the NE_L_ requirement for a 680-kg dry cow is between 14 and 15 Mcal/d. Whereas HE-fed cows consumed 52% more than their estimated NE_L_ requirement, CE consumed just 6% more than their NE_L_ requirement. However, we observed an interaction of treatment and time for prepartum NE_L_ intake (*P* = 0.05) and EB (*P* < 0.05) due to HE, and to a lesser extent CE, having greater NE_L_ intake and EB during the first 4 wk following dry-off and a decrease in NE_L_ intake and EB 2 wk before parturition. The highest mean NE_L_ intake and EB for CE and HE occurred 3 wk before parturition, at which time HE cows consumed 25.0 Mcal/d or 164% of NE_L_ requirement, whereas cows in CE consumed 18.6 Mcal/d or 121% of NE_L_ requirement. These results confirm that feeding moderate energy density diets for ad libitum intake predisposes dry cows to consume energy and nutrients well in excess of their requirements (Dann et al., 2006; Janovick and Drackley, 2010; Richards et al., 2020). In contrast, the CE diet allowed cows to consume feed at ad libitum intake, meeting the requirements for protein and other nutrients but preventing overconsumption of energy.

The HE cows gained 43.2 kg more BW than RE prepartum (*P* < 0.01), although no differences were observed between cows in CE and HE (*P* = 0.22). Greater BW gain in HE and CE was likely due to the greater DM and NE_L_ intakes, compared with cows in RE. Change in BCS did not differ (*P* ≥ 0.15) among treatments.

Despite differences in intakes of DM and NE_L_ as well as BW gain during the dry period, calf birth BW (43.8 ± 0.68 kg) did not differ (*P* = 0.81) among treatments. The estimated BW gain due to fetal growth during the dry period is 35 to 40 kg (NRC, 2001). The BW gain of cows fed HE (54.9 kg) and CE (40.7 kg) was higher than and similar to, respectively, the values estimated by NRC (2001), whereas that of RE (11.7 kg) was much less. Together with the fact that RE cows were in negative energy balance throughout the dry period, our results indicate that RE mobilized body reserves to support fetal growth. Still, changes in BCS were not detected in this group. Changes in BCS during the dry period might not be sensitive enough to reflect differences in actual body fat reserves, particularly for internal adipose depots (Drackley et al., 2014). Cows subjected to restricted feeding below requirements during the dry period lost BW and BCS in previous studies (Dann et al., 2006; Douglas et al., 2006; Colazo et al., 2009).

Postpartum DMI (*P* = 0.52), NE_L_ intake (*P* = 0.53), and EB (*P* = 0.50) did not differ among treatments (Table 2) with no interactions of treatment and time (*P* > 0.10). Others who fed lower energy diets or limited prepartum DMI reported greater postpartum DMI (Kunz et al., 1985; Holcomb et al., 2001; Agenäs et al., 2003). The lack of differences among treatments in postpartum DMI, NE_L_ intake, and EB resulted in similar (*P* > 0.10) BW and BCS (Table 2). Change in BCS was not different (*P* > 0.10) among treatments during the first 28 DIM. In contrast, previous studies from our group (Douglas et al., 2006; Janovick and Drackley, 2010; Richards et al., 2020) and other research groups (Rukkwamsuk et al., 1999; Agenäs et al., 2003; Hayirli et al., 2011) agreed that feeding moderate or high energy density diets during the dry period predisposes cows to greater BW and BCS losses after calving. This finding was true even when cows did not become over-conditioned during the dry period. Interactions of treatment and time were not detected (*P* > 0.10) for postpartum BW or BCS (Table 2).

Yields of milk, 3.5% FCM, and ECM did not differ (*P* > 0.10) among treatments (Table 3). Similarly, the contents and yields of milk components did not differ (*P* > 0.10) across treatments. No interactions of treatment and time were observed (*P* > 0.10). Similar intakes of DM and NE_L_ postpartum in our experiment may have led to the lack of differences in yields of milk, 3.5% FCM, and ECM. Huang et al. (2014) observed that feeding a high energy diet (1.63 Mcal/kg) prepartum resulted in lower milk yield during the first 70 DIM but higher FCM and milk fat concentration during the first 21 DIM compared with middle (1.48 Mcal/kg) or low (1.29 Mcal/kg) energy diets. Such responses resulted from lower DMI and higher fat mobilization that led to significant BCS loss. In agreement, Mann et al. (2015) found higher postpartum concentrations of NEFA and BHB along with a greater concentration of preformed fatty acids in milk fat in cows fed at 150% of requirement compared with cows fed to meet their NE_L_ requirements during the dry period.

**Table 3:** Least squares means and associated SEM for milk yield, milk components, and blood metabolites of cows fed controlled energy (CE), high energy (HE), or restricted energy (RE) diets during the dry period

Item	Treatment				SEM^1^	*P*-value		
	CE	HE	RE	Time		Treatment × time	CE vs. HE	HE vs. RE
Milk yield, kg/d	36.3	37.2	36.9	2.09	<0.01	0.80	0.72	0.91
3.5% FCM, kg/d	44.2	45.7	45.8	3.17	0.01	0.97	0.72	0.97
ECM, kg/d	43.2	44.3	44.2	3.04	0.01	0.95	0.78	0.99
Fat, %	5.12	5.10	5.00	0.29	0.13	0.80	0.96	0.79
Fat, kg/d	1.77	1.85	1.84	0.16	0.11	0.94	0.71	0.97
Protein, %	3.17	3.16	3.02	0.09	<0.01	0.29	0.92	0.25
Protein, kg/d	1.12	1.11	1.09	0.08	0.53	0.14	0.93	0.84
Prepartum metabolite^2^								
Glucose, mg/dL	66.5	69.8	67.8	3.46	<0.01	0.64	0.47	0.67
Insulin, μIU/mL	6.50	8.46	9.22	2.19	0.80	0.87	0.49	0.79
NEFA, mmol/L	0.56	0.50	0.57	0.12	0.01	0.68	0.68	0.67
BHB, mmol/L	0.50	0.36	0.49	0.10	0.04	0.04	0.28	0.35
Ca, mg/dL	9.18	9.21	9.08	0.23	0.01	0.95	0.93	0.66
Mg, mg/dL	2.28	2.25	2.19	0.11	0.29	0.73	0.85	0.67
Postpartum metabolite^2^								
Glucose, mg/dL	55.5	56.9	54.0	2.48	<0.01	0.27	0.67	0.39
Insulin, μIU/mL	4.69	4.50	4.98	0.89	0.18	0.06	0.87	0.69
NEFA, mmol/L	0.76	0.74	0.76	0.11	0.06	0.85	0.86	0.88
BHB, mmol/L	1.06	1.00	1.24	0.16	<0.01	0.15	0.77	0.26
Ca, mg/dL	8.55	8.73	8.55	0.20	<0.01	0.44	0.46	0.49
Mg, mg/dL	2.04	2.02	2.08	0.06	<0.01	0.43	0.79	0.48

1 Greatest SEM.

2 Blood metabolites measured from d 5 before through 5 d after calving.

Dietary treatments fed during the dry period did not affect (*P* > 0.10) pre- or postpartum blood glucose, NEFA, BHB, insulin, Ca, or Mg (Table 3). An interaction of treatment and time (*P* = 0.04) for BHB concentration prepartum was due to spikes in BHB concentration for CE and RE during d −5 to −3 before calving. The interaction of treatment and time (*P* = 0.06) for insulin concentration postpartum occurred due to the variation in insulin concentration across treatments during the 5 d postpartum. In our study, samples were taken within 1 h after feeding, which may have minimized differences among treatments.

Zimbelman et al. (2009) proposed 68 as the new upper THI threshold for modern high producing dairy cows. In our study, the high, mean, and low THI were 79.7 ± 12.3, 66.4 ± 9.4, and 57.5 ± 7.5, respectively. The high THI remained above 75 from May to September, whereas the mean THI remained above 68 from May to August. Greater mean THI occurred in months (June, July, and August) with a greater percentage of cows (41, 70, and 81%, respectively) enrolled in the study. Consequently, our cows experienced moderate to severe heat stress throughout the study, which might have caused lower DMI, NE_L_ intake, EB, and milk yield when compared with some studies (Janovick and Drackley, 2010; Mann et al., 2015). Nevertheless, no differences were noted among treatments.

In conclusion, cows fed ad libitum had greater prepartum intakes of DM and NE_L_ than cows subjected to restricted feeding. Although DMI before parturition decreased in cows fed ad libitum compared with restricted-fed cows, the decrease in NE_L_ intake was less in CE compared with HE cows. Feeding CE compared with HE at ad libitum intake throughout the dry period prevented cows from overconsuming energy relative to their requirements. Consequently, BW gain was greater in HE compared with RE-fed cows, although changes in BCS were not detected. Blood glucose, NEFA, BHB, insulin, Ca, and Mg measured 5 d before through 5 d after parturition also were not affected by dry period diet in cows that were free of health problems. Controlled energy diets did not modify peripartal metabolism in cows that remained free of metabolic disorders. Although cow numbers were limited, milk production was not affected by prepartum diets.
